# Anomalous electronic structure and magnetoresistance in TaAs_2_

**DOI:** 10.1038/srep27294

**Published:** 2016-06-07

**Authors:** Yongkang Luo, R. D. McDonald, P. F. S. Rosa, B. Scott, N. Wakeham, N. J. Ghimire, E. D. Bauer, J. D. Thompson, F. Ronning

**Affiliations:** 1 Los Alamos National Laboratory, Los Alamos, New Mexico 87545, USA

## Abstract

The change in resistance of a material in a magnetic field reflects its electronic state. In metals with weakly- or non-interacting electrons, the resistance typically increases upon the application of a magnetic field. In contrast, negative magnetoresistance may appear under some circumstances, *e.g.*, in metals with anisotropic Fermi surfaces or with spin-disorder scattering and semimetals with Dirac or Weyl electronic structures. Here we show that the non-magnetic semimetal TaAs_2_ possesses a very large negative magnetoresistance, with an unknown scattering mechanism. Density functional calculations find that TaAs_2_ is a new topological semimetal [ℤ_2_ invariant (0;111)] without Dirac dispersion, demonstrating that a negative magnetoresistance in non-magnetic semimetals cannot be attributed uniquely to the Adler-Bell-Jackiw chiral anomaly of bulk Dirac/Weyl fermions.

Magneto-transport continues to be an exciting topic in condensed matter physics. Some famous examples include discovering and understanding giant/collosal magnetoresistance^1^,^2^,^3^, integer and fractional quantum Hall effects^4^,^5^, Shubnikov-de Haas oscillations^6^, and weak localization^7^. In metals with weakly- or non-interacting electrons, the resistance typically increases upon the application of a magnetic field due to the bending of electron trajectories^8^. Negative magnetoresistance (MR) is only observed in certain circumstances. To exploit these phenomena in applications it is essential to understand the scattering mechanisms involved. Low-carrier-density systems offer an interesting platform to explore the fundamental physics of scattering processes. A recent example is SrTiO_3–*δ*_ whose *T *^2^-power law in resistivity, characteristic of a Landau Fermi liquid, cannot originate from simple electron-electron scattering, as often has been assumed^9^. Semimetals can be considered as failed semiconductors with a negative indirect band gap. Consequently, these compensated systems, with approximately equal numbers of electrons and holes, have low effective masses due to the low band filling, which leads to rich magnetotransport phenomena including extremely large positive magnetoresistance (XMR) and ultrahigh mobilities exceeding those found in giant/collosal magnetoresistance systems^1^,^2^,^3^. In addition, notions of topology have extended to semimetals as well. Accidental band crossings protected by symmetry allow electronic structures that are described by a massless Dirac equation. If either time reversal or inversion symmetry is broken, the four-fold (including spin) degenerate Dirac point splits into two Weyl points with opposite chirality. Typical examples are Cd_3_As_2_^10^ and Na_3_Bi^11^ for Dirac semimetals, and *TmPn* (*Tm* = Ta, Nb; *Pn* = As, P) for Weyl semimetals^12^. As a result of their exotic electronic structure, such semimetals host Fermi-arc surface states, XMR, Shubnikov-de Haas (SdH) oscillations, non-trivial Berry phases, and other related phenomena^13^,^14^,^15^,^16^,^17^,^18^,^19^,^20^. Importantly, Dirac/Weyl semimetals are expected to have negative magnetoresistance when current is parallel to a magnetic field due to the Adler-Bell-Jackiw (ABJ) chiral anomaly mechanism^21^,^22^,^23^,^24^. The ABJ anomaly is a consequence of the chemical potential changing at each of the Weyl nodes, giving rise to an additional conduction channel, and has been taken as a smoking gun for the existence of a Dirac and/or Weyl semimetal.

If no accidental band crossings occur, can one still consider a semimetal as topologically non-trivial? The answer is yes. Similar to the classification for band insulators, 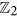 topological indices (*v*_0_;* v*_1_*v*_2_*v*_3_) (strong and weak) are still appropriate for a regular semimetal due to the presence of a continuous energy gap between electron-like and hole-like bands. The surface states associated with weak (strong) topological indices are expected to be sensitive (immune) to disorder. Herein, we investigate a novel non-magnetic semimetal TaAs_2_ that is homologous to the OsGe_2_-type crystalline structure^25^ respecting inversion symmetry (Fig. 1a). Magnetotransport measurements manifest a nearly compensated semimetal with low carrier density (~10^19^ cm^−3^), high mobility (~10^3^ cm^2^/Vs) and unsaturated XMR (~4,000,000% at 65 T and 0.5 K). Further, angular dependent longitudinal magnetoresistance (LMR) measurements show pronounced negative MR (~−98%), which suggests involvement of a ABJ chiral anomaly. Our first-principles calculations based on Density Functional Theory (DFT) confirm the semimetallicity of TaAs_2_ but finds no evidence for a Dirac-like band-crossing. Instead, by computing the 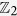 indices, (0;111), TaAs_2_ is found to be a “weak” topological material in all three reciprocal lattice directions but not a “strong” topological material. Consequently, TaAs_2_ should host surface states due to its electronic topology. We suggest that the very large negative magnetoresistance is a consequence of this novel topological state. Our observation of negative LMR in TaAs_2_ also illustrates that the scattering mechanisms in (topological) semimetals are still not sufficiently understood.

## Results

**Figure 1:**
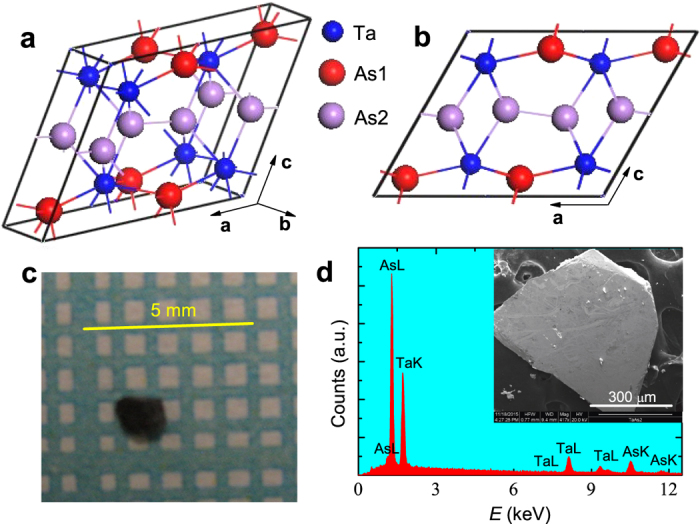
Crystalline structure of TaAs_2_ and sample characterization. (**a**) Crystalline structure of TaAs_2_. (**b**) A side view of TaAs_2_ along (010)-axis. (**c**), A photograph of TaAs_2_ single crystal on millimeter-grid paper. (**d**), A representative EDS spectrum of TaAs_2_. The inset shows the SEM image of the same sample.

Figure 1a shows the crystalline structure of TaAs_2_. It crystallizes in a monoclinic structure with space group C12/m1 (No. 12, symmorphic). There are two chemical sites for As atoms in each unit cell, labeled As1 and As2, respectively. As1 and Ta form Ta-As planes. The interlayer coupling is bridged by As2 atoms, which reside near the central plane along the **c**-axis (see Fig. 1b). Each Ta atom has eight nearest neighbors: five As1 and three As2. Figure 1c shows a TaAs_2_ single crystal with a typical size on millimeter-grid paper. EDS analysis gives the mole ratio Ta:As = 1:1.90(5), within experimental error consistent with the stoichiometric ratio. By XRD refinement, we deduce the crystalline lattice parameters listed in Table 1. Most importantly, inversion symmetry is respected in this compound.

**Figure 2:**
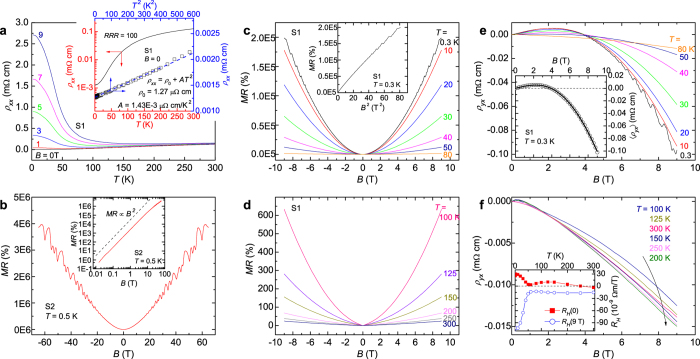
Transport properties of TaAs_2_. (**a**) Temperature dependencies of 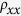 at selected magnetic fields. The inset shows *RRR* and Fermi liquid behavior at *B* = 0. (**b**) Unsaturated *MR* up to 65 T at 0.5 K. The inset demonstrates quadratic-like MR(*B*). (**c**,**d**) field dependent *MR* at various temperatures. The inset to **c** shows *MR* vs. *B*^2^ at 0.3 K. (**e**,**f**) Field dependent 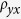 at various temperatures. The inset to (**e**) displays a two-band fit of 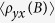 at 0.3 K. The inset to **f** displays the Hall coefficient *R*_*H*_ as a function of *T*.

In the absence of magnetic field, TaAs_2_ shows a metallic Fermi-liquid-like *ρ*_*xx*_(*T*) profile, with a large residual resistivity ratio *RRR *≡ *ρ*_*xx*_(300 K)/*ρ*_*xx*_(0.3 K) ≈ 100 (inset to Fig. 2a), manifesting good sample quality. There is no signature of superconductivity above 0.3 K. When a magnetic field is applied, *ρ*_*xx*_(*T*) turns up and exhibits insulating-like behavior before it levels off at low temperature. Similar behavior is observed in other semimetallic materials^26^,^27^,^28^. The insulating-like behavior becomes more and more pronounced as field increases, which leads to an XMR at low temperature. In Fig. 2b, we show *MR*(*B*)[≡(*R*(*B*) − *R*(0))/*R*(0) × 100%] measured at 0.5 K and in fields up to 65 T. The *MR* reaches ~4,000,000% (~200,000%) at 65 T (9 T), without any signature of saturation. Unlike the linear or sub-linear *MR*(*B*) observed in the Dirac semimetal Cd_3_As_2_^29^ and the Weyl semimetals *TmPn*^15^,^16^,^18^,^19^, here *MR*(*B*) generally obeys a parabolic field dependence (inset to Fig. 2c), although the exponent decreases slightly at very high field (inset to Fig. 2b). Such behavior is reminiscent of WTe_2_^28^, a candidate type-II Weyl semimetal^30^.

In Fig. 2e,f, we present Hall effect data. For all temperatures measured, the field-dependent Hall resistivity 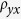 is strongly non-linear and changes from positive at low field to negative at high field. The non-linearity of 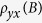 is reflected further by the divergence between the Hall coefficients *R*_*H*_(0) and *R*_*H*_(9 T) as shown in the inset to Fig. 2f. Here, *R*_*H*_(9 T) is defined by 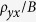 at *B *= 9 T, and *R*_*H*_(0) is the initial slope of 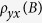 near *B *= 0. All these features are characteristic signatures of multi-band effects. Indeed, 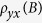 can be well fit to a two-band model,


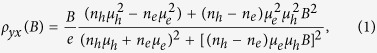


where *n* and *μ* are respectively carrier density and mobility, and the subscript *e* (or *h*) denotes electron (or hole). A representative fit to 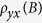 at *T* = 0.3 K is shown in the inset to Fig. 2e, and from this fit we obtain *n*_*e *_= 1.4(2) × 10^19^ cm^−3^, *n*_*h *_= 1.0(1) × 10^19^ cm^−3^, *μ*_*e*_ = 1.9(2) × 10^3^ cm^2^/Vs, and *μ*_*h*_ = 2.5(2) × 10^3^ cm^2^/Vs. The carrier densities are close to those estimated from the analysis of SdH oscillations [see Supplementary Information (*SI*) *II*]. The low carrier density confirms TaAs_2_ to be a semimetal. Furthermore, the imbalance between *n*_*e*_ and *n*_*h*_ implies that it is not a perfectly compensated semimetal^31^.

**Figure 3:**
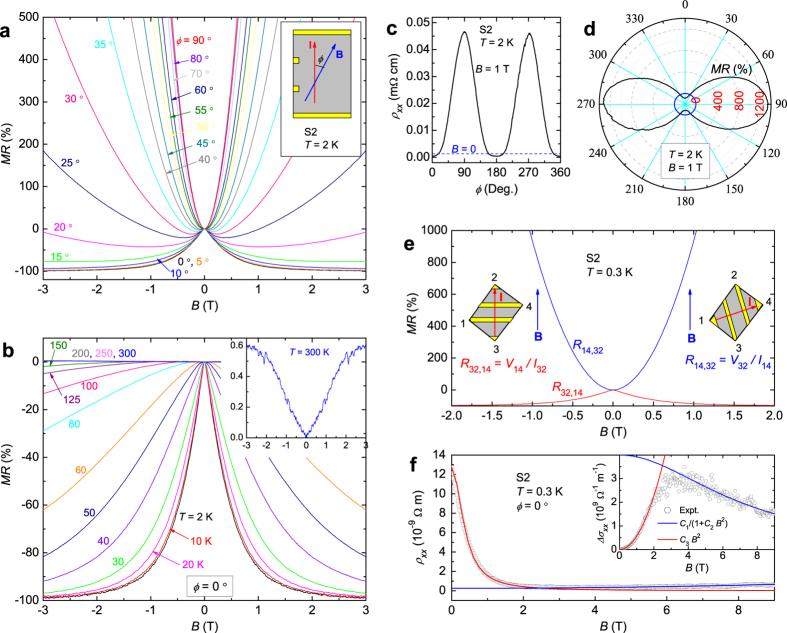
Longitudinal magnetoresistance (LMR) of TaAs_2_. (**a**) Field-dependent *MR* of TaAs_2_ with various angles *ϕ* at 2 K. The inset shows the configuration of the measurements. (**b**) *MR* at different temperatures, measured at *ϕ* = 0. The inset displays the data at 300 K. (**c**) The angular dependence of 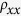 at 2 K and 1 T. (**d**) A polar plot of *MR* at 2 K. (**e**) MR with two different measurement geometries, *R*_32,14_ = *V*_14_/*I*_32_ (red) and *R*_14,32_ = *V*_32_/*I*_14_ (blue). Schematic sketches of the geometry are shown in the insets. (**f**) Theoretical fits of 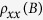 and 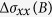. The high-field part of 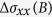 is fit to 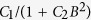 (blue), and the low-field part is fit to 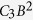 (red). The measurements were done in the contact geometry as shown in the inset to (**a**).

One important feature of topological Dirac/Weyl semimetals is the so-called ABJ chiral anomaly^23^,^24^. The ABJ anomaly is a result of chiral symmetry breaking when *B·E* is finite. This gives rise to a charge-pumping effect between opposite Weyl nodes. An additional contribution to the total conductivity is generated, *i.e.*, *σ*_**x**_ ∝ *B*^2^, observable as a negative LMR^20^,^24^. In Fig. 3a, we present the *MR*(*B*) at 2 K and various *ϕ* (*ϕ* is the angle between **B** and electrical current **I**). Indeed, we observe a striking negative LMR when *ϕ* = 0. The *MR* reaches −98% before it starts to turn up weakly at high field (Fig. 3f), which we ascribe to a small angular mismatch (see below). The negative LMR also persists to high temperatures *T * > 150 K (cf Fig. 3b). Compared with the chiral-anomaly-induced negative LMRs observed in Dirac/Weyl semimetals, such as Na_3_Bi^20^ and *TmPn*^15^,^19^, the one seen in TaAs_2_ is bigger in magnitude and survives at much larger *ϕ* and higher *T*. For example, Fig. 3c plots 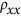 measured at 1 T and 2 K as a function of *ϕ*, and the angular dependent *MR* is sketched in a polar plot in Fig. 3d. Clearly, the negative LMR survives for *ϕ* as large as 30°. Note that the cusp near *B* = 0 is not overcome until *ϕ *> 45 (Fig. 3a). In contrast to other systems^15^,^19^,^20^, because 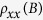 increases as *B*^2^ when **B⊥I**, the slow rate of increase in MR in the vicinity of zero field makes it more robust against angular mismatch. This also allows the negative LMR in the limit of *B* → 0. Taking only 2% residual resistivity at 3 T and the total carrier density *n*_*t*_( = *n*_*e*_ + *n*_*h*_) = 2.4 × 10^19^ cm^−3^, we estimate the average transport mobility 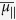 = 1.0 × 10^7^ cm^2^/Vs. Using the Fermi-surface parameters of the electron-pocket as an example (see *SI II*), we further calculate the Fermi velocity *v*_*F*_ = 7.9 × 10^5^ m/s, and transport relaxation time *τ* = 4.8 × 10^−10^ s. This means that the carriers can travel a distance (viz. mean free path) *l* = 0.4 mm without backward scattering. Such an anisotropic MR and field induced low-scattering state would apparently find applications in electronic/spintronic devices, but the scattering mechanism is an open question.

**Figure 4:**
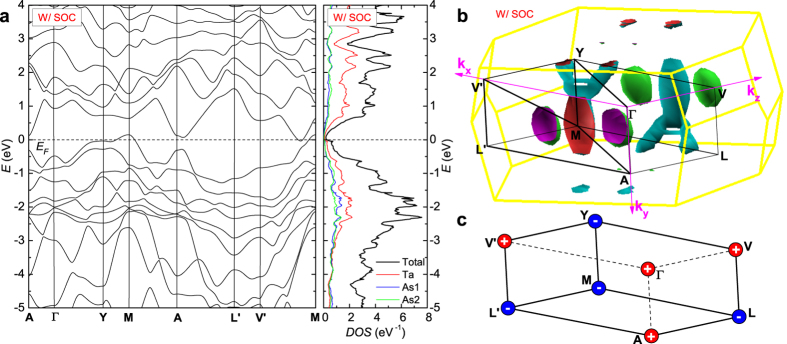
DFT calculations of TaAs_2_ with SOC. (**a**) Band structure and DOS of TaAs_2_. (**b**) FS topology and TRIM points. (**c**) Parity of the TRIM at the monoclinic Brillouin zone.

Figure 4a shows the band structure and density of states (DOS) calculated with spin-orbit coupling (SOC). The semimetallic character can be seen by the low DOS at the Fermi level and the presence of small electron- and hole-bands. Figure 4b shows the Fermi surface (FS) topology calculated with SOC. The FS of TaAs_2_ mainly consists of one hole- and two electron-pockets. The electron-pockets, located off the symmetry plane, are almost elliptical. The hole-pocket encompasses the **M** point at (1/2, 1/2, 1/2) but is more anisotropic with two extra “legs”. The abnormal FS structure of the hole pocket also is reflected in the complicated SdH frequencies discussed in the *SI II*. Two additional electron-like pockets with vanishingly small size are observed intersecting the top of the Brillouin zone. Without SOC, accidental band crossings do occur as shown in the *SI III*, and they can be classified as type-II Dirac points^30^. Upon adding SOC, however, these Dirac points become gapped, and a careful survey over the entire Brillouin zone reveals no accidental band crossings in the vicinity of the Fermi level. The possibility of a Weyl semimetal is in any event excluded due to the preservation of both time reversal and inversion symmetries.

## Discussion

Due to the continuous gap in the band structure, the 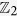 indices can be computed. The presence of inversion symmetry allows us to compute the topological indices (*v*_0_;* v*_1_*v*_2_*v*_3_) based only on the parities of the occupied wave functions at time-reversal-invariant-momenta (TRIM)^32^. The results are shown in Fig. 4c. (Refer to *SI IV* for more details.) The unoccupied states of the hole band at **M** do not influence the topological indices because these states have even parity. The product of parities over all the TRIM gives the value of the so-called “strong” topological index *v*_0_. As can be seen from Fig. 4c, the electronic structure is trivial from this perspective. Nevertheless, all three “weak” topological indices (*v*_1,2,3_) are non-trivial. Hence, surface states are mandated by these weak topological indices, although they are believed to be sensitive to disorder.

We now return to the issue of the negative LMR. An electric current parallel to a magnetic field is not expected to experience a Lorentz force; however, in reality, negative LMR may exist stemming from a variety of mechanisms. First, because TaAs_2_ is non-magnetic, a magnetic origin can be ruled out. Second, weak localization is also excluded, because 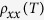 conforms to Fermi-liquid behavior at low temperatures, and no −log*T* or any form of upturn signature can be identified. Third, negative LMR was also observed in materials such as PdCoO_2_^33^ with high FS anisotropy. To test the role of FS anisotropy, we measured the magnetoresistances of *R*_32,14_ and *R*_14,32_ with the schemes shown in the insets to Fig. 3e. In the measurements of *R*_32,14_, the current is parallel to **B**, and we derived a negative LMR, but the MR of *R*_14,32_ is positive. Similar results were reproduced on several other samples with different shapes. Because the direction of current is arbitrary when referenced to the crystalline axes, these measurements imply that the observed negative LMR is locked to the relative angle between **E** and **B**, rather than pinned to particular FS axes. Fourth, an improperly made contact geometry may also cause negative LMR especially when the material shows a large transverse MR, known as the “current-jetting” effect^8^,^34^,^35^,^36^. We have performed a series of careful LMR measurements with different contact geometries, and the results reveal that albeit a current-jetting effect can occur, the large negative LMR, however, is also intrinsic. *SI V* provides more details.

To study further the features of this negative LMR, we plot Δ*σ*_*xx*_(*B*) = *σ*_*xx*_(*B*) − *σ*_*xx*_(0) =  in the inset to Fig. 3f. The low field part of Δ*σ*_*xx*_ can be well fitted to the form *C*_3_*B*^2^ (red line), which is consistent with an ABJ chiral conductivity *σ*_**x**_. The absence of Dirac or Weyl points in TaAs_2_, however, indicates that the negative LMR is not a consequence of the ABJ chiral anomaly as has been posited for other Dirac and Weyl semimetals. The fitting is converted back to 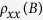 as shown in the main frame of Fig. 3f (red line). At high field, this fitting is gradually violated due to the emergence of a weak parabolic term in 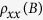, for which we successfully fitted 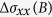 to the formula 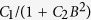 (blue line). This weak positive MR is probably due to a small angular mismatch that causes a parabolic *MR*(*B*) which becomes dominant as field strengthens (See also in *SI V*).

Given the absence of alternative possibilities, an interesting question is whether the presence of topological surface states coexisting with a bulk semimetallic electronic structure could produce the large negative LMR as we observe. We note that conductivity corrections are found when surface states interact with bulk conduction states^37^, although the observed effect here is an increase in the conductivity of a factor of ~50. Having ruled out possible interpretations for the origin of a firmly established large, negative LMR in TaAs_2_, this work calls for future theoretical and experimental work.

In summary, we find that single crystals of TaAs_2_ grown by vapor transport are semimetals with extremely large, -unsaturating transverse magnetoresistance characteristic of high mobilities. Strikingly, TaAs_2_ hosts a negative longitudinal magnetoresistance that reaches −98%. TaAs_2_ is an example of a semimetal whose strong topological index is trivial, yet all three of its weak topological indicies are non-trivial. Similar properties also may exist in other OsGe_2_-type *TmPn*_2_ compounds where *Tm* = Ta and Nb, and *Pn* = P, As and Sb. As was the case for giant magnetoresistance, potential applications exist if the scattering mechanisms in these semimetals can be understood and manipulated.

*Note added: When completing this manuscript, we became aware of several other related works*^38^,^39^,^40^,^41^.

## Methods

### Sample synthesis and characterization

Millimeter-sized single crystals of TaAs_2_ were obtained as a by-product of growing TaAs by means of an Iodine-vapor transport technique with 0.05 g/cm^3^ I_2_. First, polycrystalline TaAs was prepared by heating stoichiometric amounts of Ta and As in an evacuated silica ampoule at 973 K for three days. Subsequently, the powder was loaded in a horizontal tube furnace in which the temperature of the hot zone was kept at 1123 K and that of the cold zone was ~1023 K. Several TaAs_2_ single crystals with apparent monoclinic shape were picked from the resultant and their monoclinic structure^25^ and stoichiometry were confirmed by x-ray diffraction (XRD) and energy dispersive x-ray spectroscopy (EDS). No I_2_ doping was detected, and the stoichiometric ratio is fairly homogenous.

### Measurements

Three TaAs_2_ single crystals (labeled S1, S2 and S3) were polished into a plate with the normal perpendicular to the ab-plane. Ohmic contacts were prepared on the crystal in a Hall-bar geometry, and both in-plane electrical resistivity (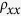) and Hall resistivity (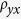, S1 only) were measured by slowly sweeping a DC magnetic field from −9 T to 9 T at a rate of 0.2 T/min. 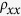 (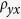) was obtained as the symmetric (antisymmetric) component under magnetic field reversal. An AC-resistance bridge (LR-700) was used to perform these transport measurements in a 3-He refrigerator. Field-rotation measurements were carried out using a commercial rotator on a Physical Property Measurement System (PPMS-9, Quantum Design). Different contact geometries were made on S3 to show a possible current-jetting effect, and the measurements were performed in a 3-axis magnet. Magnetoresistance also was measured up to 65 T in a pulsed field magnet at the National High Magnetic Field Laboratory (NHMFL, Los Alamos). Several additional samples with different shapes were measured to confirm the reproducibility of negative LMR.

### DFT calculations

Density functional theory calculations were performed using the generalized gradient approximation (GGA) as implemented in the WIEN2K code^42^ with the exchange correlation potential of Perdew-Burke-Ernzerhof (PBE)^43^. Spin-orbit coupling on all atoms without relativistic local orbitals was included in a second variational scheme. The structure of TaAs_2_ was obtained from Rietveld refinement (Table 1).

## Additional Information

**How to cite this article**: Luo, Y. *et al.* Anomalous electronic structure and magnetoresistance in TaAs_2_. *Sci. Rep.* **6**, 27294; doi: 10.1038/srep27294 (2016).

## Supplementary Material

Supplementary Information

## Figures and Tables

**Table 1:** Crystalline lattice parameters of TaAs_2_.

Atoms	*x*	*y*	*z*	Occ.
Ta	0.1574	0	0.1959	1.00
As1	0.4058	0	0.1072	1.00
As2	0.1394	0	0.5260	1.00

Space group: C2/m1 (No. 12). *a *= 9.370 Å, *b *= 3.394 Å, *c *= 7.771 Å, *α* =  *γ *= 90°, and *β *= 119.725° . The atomic positions (*x*, *y*, *z*) are listed above.
