# Supplementary material for: Anomalous electronic structure and magnetoresistance in TaAs2
Source: Sci Rep. 2016 Jun 7;6:27294. doi: 10.1038/srep27294 (PMC4895157; doi:10.1038/srep27294)
Supplement: Supplementary Information [file srep27294-s1.pdf]

Supplementary Information for:

Anomalous electronic structure and magnetoresistance in $TaAs_2$

Yongkang Luo*, R. D. McDonald, P. F. S. Rosa, B. Scott, N. Wakeham,

N. J. Ghimire†, E. D. Bauer, J. D. Thompson‡, and F. Ronning§

[1] *Los Alamos National Laboratory, Los Alamos, New Mexico 87545, USA.*

(Dated: April 17, 2016)

* Electronic address: ykluo@lanl.gov

† Present address: Materials Science Division, Argonne National Laboratory, Argonne, Illinois 60439, USA

‡ Electronic address: jdt@lanl.gov

§ Electronic address: fronning@lanl.gov

In this ***Supplementray Information*** (***SI***), we provide magnetization, Shubnikov-de Hass (SdH) quantum oscillations, additional Density-functional-theory (DFT) calculations, topological indices, and longitudinal magnetoresistance (LMR) that further support the discussion and conclusions of the main text.

SI I: Magnetization of TaAs$_2$

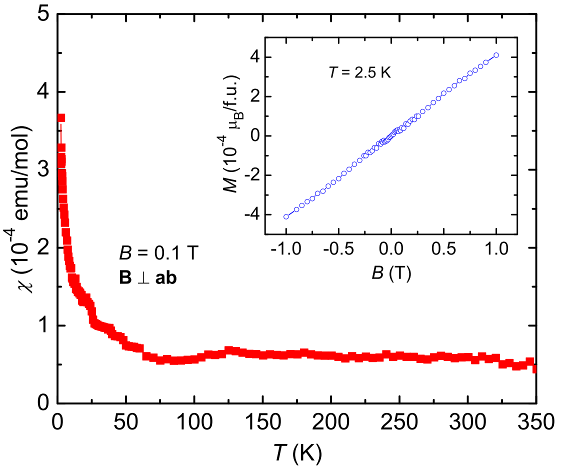

Figure S1 | ***Magnetization of TaAs$_2$.*** *Main frame, temperature dependence of magnetic susceptibility χ. The inset shows isothermal field dependent magnetization at 2.5 K.*

The magnetic properties of TaAs$_2$ are displayed in Figure S1. These measurements were taken on sample S1 with an external magnetic field **B**$\perp$**ab**, using a Quantum Design Magnetic Property Measurement System (MPMS-5). The main frame of Figure S1 shows the temperature dependence of magnetic susceptibility $\chi(T)$. The value of χ is 5.94$\times$10^{-5} emu/mol at room temperature, and remains essentially unchanged down to 50 K. The weak upturn at low temperature is likely an impurity contribution. Such a Pauli-paramagnetic $\chi(T)$ curve can not be described by the well-known Curie-Weiss law, manifesting the absence of intrinsic local moments. This is further confirmed by the isothermal field dependent magnetization at 2.5 K as shown in the inset to Figure S1. The magnetization reaches only 4$\times$10^{-4} μ_B/f.u. at 1 T. All these results demonstrate that TaAs$_2$ is a non-magnetic compound, and the large transverse magnetoresistance and

negative longitudinal magnetoresistance discussed in the main text do not have a magnetic origin. In addition, we may also conclude that time reversal symmetry is respected in $TaAs_2$.

SI II: SdH oscillations

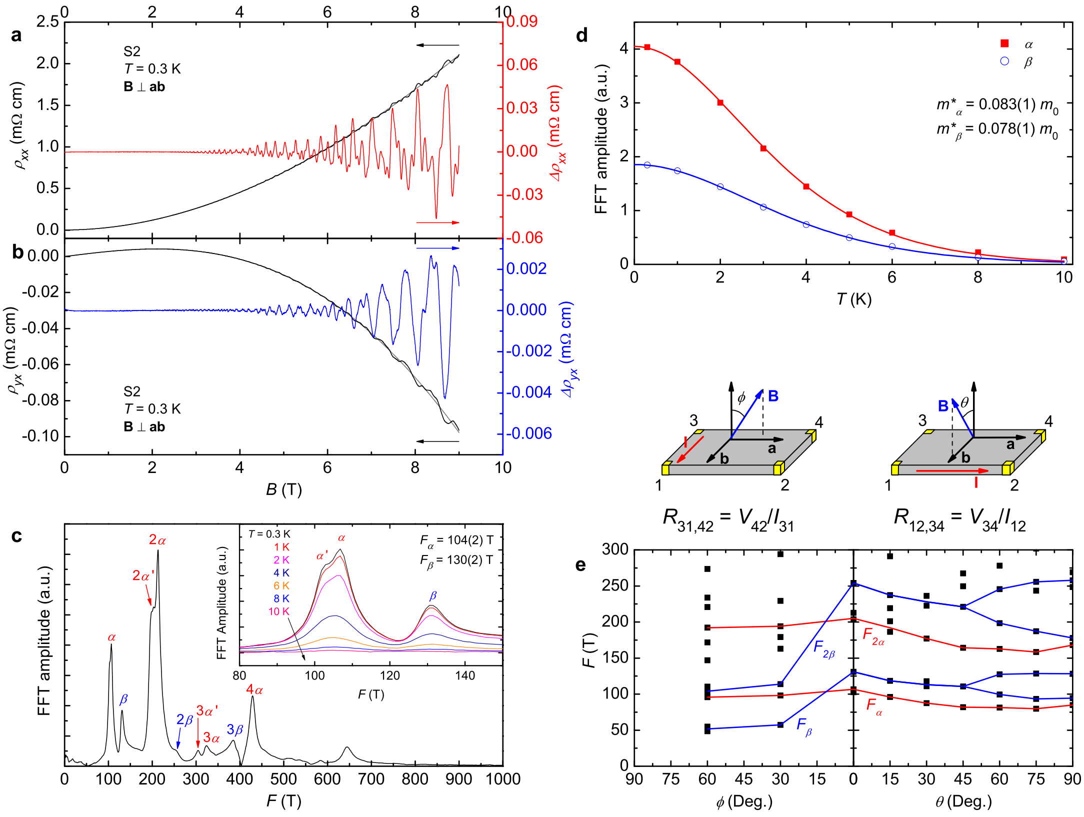

Figure S2* | *SdH quantum oscillations of $TaAs_2$. ***a*** *and* ***b****, Field dependence of* ρ_{xx} *and* ρ_{yx} *at 0.3 K for* $\mathbf{B}\perp\mathbf{ab}$*. The grey lines are the fourth-order polynomial fitting. The right axes show oscillatory contributions,* $\Delta\rho_{xx}$ *and* $\Delta\rho_{yx}$*, respectively.* ***c****, FFT spectrum of* $\Delta\rho_{xx}(1/B)$*. The inset is a zoom-in view at selected temperatures.* ***d****, Temperature dependent FFT amplitudes of* α*- and* β*-pockets. Fitting to the LK formula results in effective masses* m^*_α*=0.083(1)* m_0*,* m^*_β*=0.078(1)* m_0*. The solid lines are theoretical fittings to LK formula.* ***e****, Angular (*θ *and* ϕ*) dependence of FS cross-sectional extrema. The solid lines are guidelines. The scheme of the measurements is also depicted.*

Another important feature of the magnetotransport property of $TaAs_2$ is the SdH quantum oscillation atop of the large magnetoresistance signal. The analysis of SdH data is somewhat complicated. Further systematic measurements under higher magnetic field are needed to better clarify the details of the Fermi surface (FS) topology. We leave this task for future work. To estimate the carrier density we analyse the SdH oscillations based on our angular measurements up to 9 T.

The SdH effect can be observed in both $\rho_{xx}(B)$ (Figure S2a) and $\rho_{yx}(B)$ (Figure S2b). We derive the oscillatory part from $\Delta\rho_{ij}$=ρ_{ij}−$\langle\rho_{ij}\rangle$ (i,j=x,y), where the non-oscillatory part $\langle\rho_{ij}\rangle$ is obtained by a fourth-order polynomial fit to $\rho_{ij}(B)$. The obtained $\Delta\rho_{xx}(B)$ and $\Delta\rho_{yx}(B)$ are displayed with the right axes of Figure S2a and S2b, respectively.

By taking the Fast Fourier Transformation (FFT) of $\Delta\rho_{xx}$ as a function of $1/B$, we obtain multiple SdH oscillation frequencies as shown in Figure S2c. The two fundamental frequencies are F_α=104(2) T and F_β=130(2) T. The decaying amplitude of SdH oscillations with temperature is described by the Lifshitz-Kosevich (LK) formula[1]:

$$\frac{\Delta\rho_{xx}}{\langle\rho_{xx}\rangle} \propto \frac{2\pi^2 k_B T/\hbar\omega_c}{\sinh(2\pi^2 k_B T/\hbar\omega_c)}, \tag{S1}$$

in which ω_c=$\frac{eB}{m^*}$ is the cyclotron frequency with m^* being the effective mass. We tracked the FFT amplitudes of α- and β-orbits as a function of T in Figure S2(d). Fitting these data points to the LK formula, we derived the effective masses, m^*_α=0.083(1) m_0 and m^*_β=0.078(1) m_0, where m_0 is the mass of a free electron. These small effective masses are similar to those in other topological materials, *e.g.*, 0.089 m_0 for the 3D topological insulator Bi_2Te_2Se[2, 3], 0.043 m_0 for the Dirac semimetal Cd_3As_2[4] and 0.033-0.066 m_0 for the Weyl semimetal NbAs[1], but are much smaller than those of $NbSb_2$ ($\sim$1 m_0), an iso-structural analog[5], and for WTe_2 ($\sim$0.4 m_0)[6], a candidate type-II Weyl semimetal[7].

We also performed angular dependent SdH oscillation measurements. θ and ϕ respectively depict the angles between electrical current **I** and magnetic field **B** when the field is rotated in two different ways, see Figure S2e. The oscillatory frequencies are shown in Figure S2e, and the possible guidelines for their angular dependencies are also presented. Combining this with the DFT calculations addressed in the main text, we may assume the α-orbit is due to an electron-pocket, and the β-orbit is due to a hole-pocket.

For a three dimensional system, the carrier density is correlated with the size of Fermi

surface via,

$$n = \frac{g k_F^x k_F^y k_F^z}{3\pi^2} = \frac{g}{3\pi^2}\sqrt{\frac{8e^3 F_x F_y F_z}{\hbar^3}}, \tag{S2}$$

where g is the multiplicity of the Fermi surface in the first Brillouin zone, $k_F^i(i{=}x,y,z)$ is the magnitude of Fermi momentum along **i**-axis, and F_i is the oscillatory frequency with magnetic field **B** parallel to **i**-axis. Here, we have included the spin degeneracy and adopted the Lifshitz-Onsager correlation $F{=}\frac{\hbar}{2\pi e}S_F$, in which S_F is the extremal cross-sectional area of the Fermi surface. As a rough estimate, we treat the electron FS as a spherical pocket, with $F_{\alpha x}{=}F_{\alpha y}{=}F_{\alpha z}{\approx}100$ T and $g_e{=}2$. The carrier density of electrons is thus calculated $n_e{=}1.1{\times}10^{19}$ cm^{-3}. Due to the complex Fermi surface topology, we are not able to calculate the carrier density of holes directly, but considering the magnitude of oscillation frequency and the multiplicity $g_h{=}1$, it is reasonable to set n_e as the upper limit of n_h. These estimates are quantitatively in agreement with the Hall effect measurement discussed in the main text.

Finally, it should be pointed out that the observed F_α peak weakly splits into two at low temperature, labeled as α and α' (cf the inset to Figure S2c). This is better seen in their higher-order harmonics. This splitting seems to disappear as field rotates. It is likely that this splitting is caused by a certain sub-structure on the FS which results in additional cross-sectional extrema at a particular angle. F_β shows a much stronger angular dependence (Figure S2e); however, this might be not surprising considering its complicated topology. More systematic angular SdH measurements under higher magnetic field are required to further resolve this.

SI III: Additional DFT calculations

In Figure S3, we present additional DFT calculation results. Figure S3a shows the band structure and density of states (DOS) calculated without spin-orbit coupling (SOC) along the same path as in Figure 4 of the main text where SOC is included. Two extra cuts, **K-M** and **K'-K"**, as indicated in Figure S3b, are presented in detail to illustrate the effects of SOC. A small gap of ~60 meV between conduction- and valence-bands is observed along **K'-K"**. More interestingly, a band-crossing occurs along **K-M**. However, in contrast to regular Dirac/Weyl semimetals, the band-crossing appears at the contact of electron- and hole-pockets, rather than a point-like FS (viz. type-I Dirac/Weyl point). This reminds

us of the type-II Dirac/Weyl semimetal proposed by Soluyanov *et al* recently[7]. However, this crossing is not protected by the symmetry of the crystal lattice. Hence, when SOC is included the Dirac-like point becomes gapped. The comparisons between band structures without and with SOC along the two cuts are shown in Figure S3c and S3d, respectively. When SOC is turned on, the band crossing along **K-M** opens a small gap with magnitude ∼117 meV. Intuitively, the band gap in **K'-K"** is further enlarged by SOC.

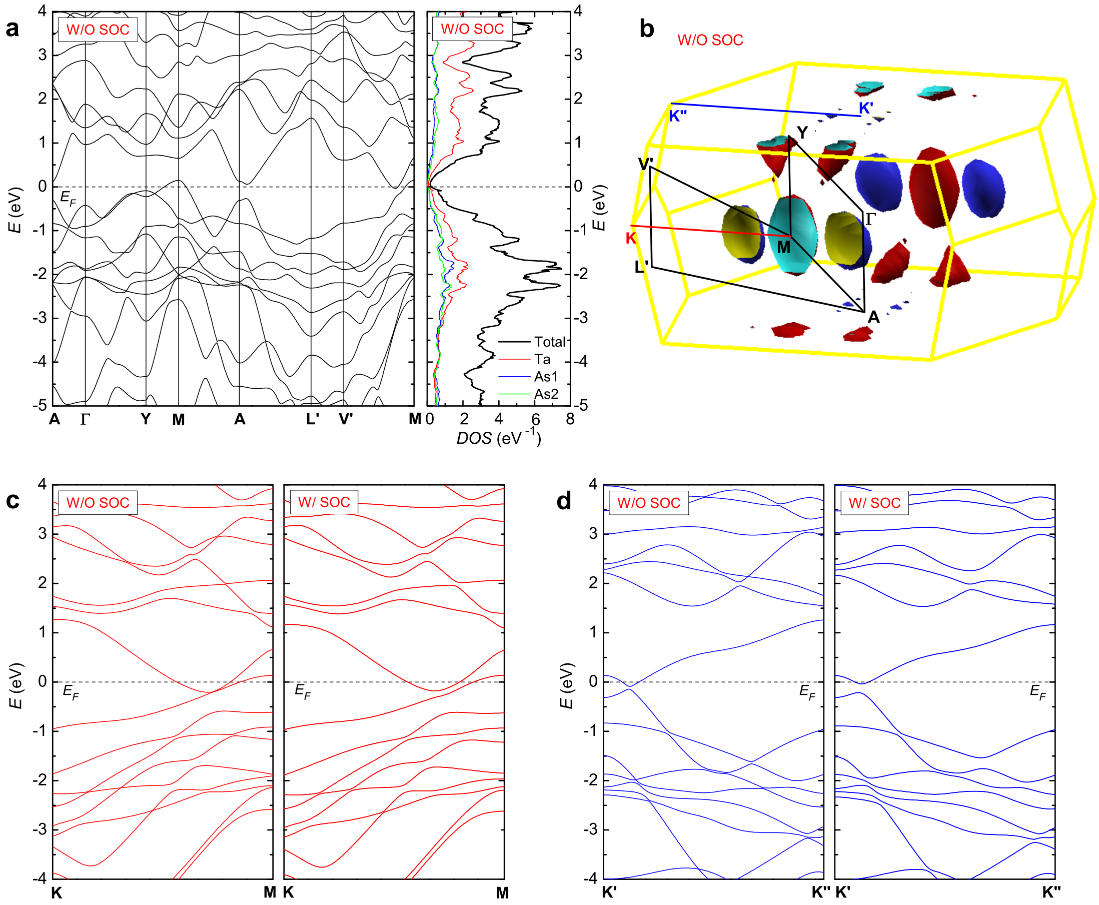

Figure S3* | *DFT calculations of $TaAs_2$ without SOC.* a**, *Band structure and DOS of $TaAs_2$ calculated without SOC.* ***b, *FS topology with the* ***k****-path cuts used in panels* ***c*** *and* ***d****.* ***c*** *and* ***d*** *respectively display the comparisons of band structure along* ***K-M*** *and* ***K'-K"*** *with and without SOC.*

SI IV: $\mathbb{Z}_2$ topological invariants

The DFT calculations also allow us to analyse the $\mathbb{Z}_2$ topological invariants (ν_0;$\nu_1\nu_2\nu_3$) for a centrosymmetric crystal[8]. Given the parity eigenvalue of the pair of occupied degenerate bands, we determine the parity (δ) of the each time-reversal-invariant-momentum (TRIM) points in the BZ as summarized in Table S1 (see also in Figure 4c).

Table S1: Parity of the TRIM of the monoclinic Brillouin zone. *Calculated based on DFT with SOC turned on.*

TRIM	(k_x, k_y, k_z)	δ	TRIM	(k_x, k_y, k_z)	δ
$\mathbf{\Gamma}$	(0, 0, 0)	+1	**A**	(0, 1/2, 0)	+1
**Y**	(1/2, 0, 1/2)	−1	**M**	(1/2, 1/2, 1/2)	−1
**V**	(0, 0, 1/2)	+1	**L**	(0, 1/2, 1/2)	−1
$\mathbf{V}'$	(1/2, 0, 0)	+1	$\mathbf{L}'$	(1/2, 1/2, 0)	−1

A sign change of δ between two TRIM points manifests a band inversion. The strong topological index ν_0 is defined by $(-1)^{\nu_0}=\prod \delta_i$, where $\prod$ goes through all the eight TRIM points. This leads to ν_0=0. The weak topological indices are calculated via the product of δ_i at four coplanar TRIM points in the BZ[9], *i.e.*, $(-1)^{\nu_1}=\delta_\mathbf{M}\delta_\mathbf{Y}\delta_{\mathbf{V}'}\delta_{\mathbf{L}'}$, $(-1)^{\nu_2}=\delta_\mathbf{M}\delta_{\mathbf{L}'}\delta_\mathbf{A}\delta_\mathbf{L}$, $(-1)^{\nu_3}=\delta_\mathbf{M}\delta_\mathbf{L}\delta_\mathbf{V}\delta_\mathbf{Y}$, and we obtain $\nu_1=\nu_2=\nu_3=1$. This analysis suggests that $TaAs_2$ is a "weak" topological material in all three reciprocal lattice directions, but not a "strong" topological material.

SI V: Additional LMR

Improperly made contact geometry may also cause negative LMR, especially when the material shows a large transverse MR, a so-called "current-jetting" effect[10–12]. To test if this will affect the LMR we have observed in $TaAs_2$, we performed a series of measurements with various contact geometries, and results are summarized in Figure S4. These measurements were done in a 3-axis magnet ($B_{x,y,z}$) to precisely tune the field orientation. We start with the case of inhomogeneous current as shown in Figure S4a, from which we indeed

obtain significantly different MR behaviors in R_A=V_{34}/I_{12} and R_B=V_{56}/I_{12}. Although R_B decreases rapidly with increasing B_z, the MR of R_A is dramatically positive. It might not be too surprising for such a divergence in the context of a current-jetting effect: due to the large transverse MR, the path 1-5-6-2 becomes more and more resistive under magnetic field, and therefore more and more current is accumulated along the path 1-3-4-2, which inevitably increases (decreases) the voltage drop between the electrodes 3 (5) and 4 (6).

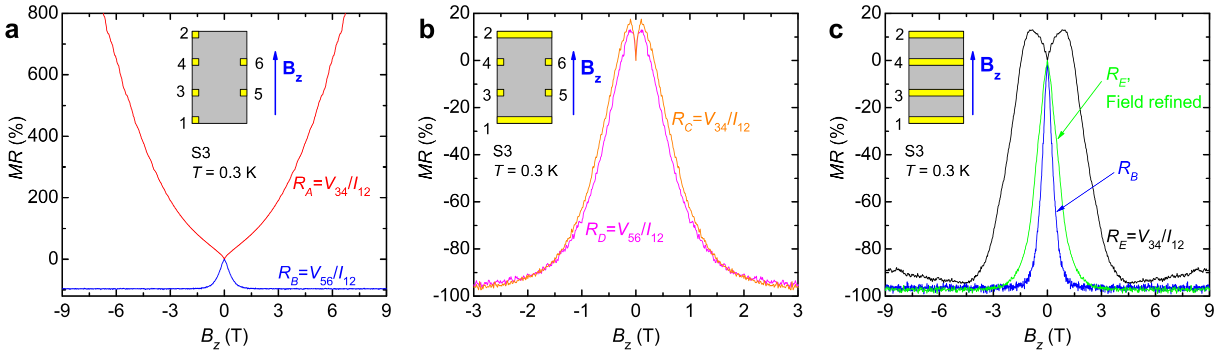

Figure S4* | *LMR measurements with various contact geometries. ***a***, *An inhomogeneous current indeed causes different magnetoresistance behaviors in* R_A=V_{34}/I_{12} *and* R_B=V_{56}/I_{12}. ***b***, *Measurements in a Hall-bar geometry. Negative LMR is seen in both* R_C *and* R_D. ***c***, *A four-probe measurements* (R_E) *with all the contacts fully across the width. A small positive MR is seen on top of the large negative LMR. Intrinsic negative LMR can be seen after the field direction is carefully tuned.*

To avoid this current-jetting effect, we improved the current homogeneity by fully painting the current leads across the end faces of the sample (Figure S4b). The derived resistances are now labeled R_C and R_D, respectively. Both of R_C and R_D exhibit negative LMR although in a small region near B_z=0 the MR initially turns up. We attribute this small positive MR to a angular mismatch (see below). It should be pointed out that the sample was installed on a home-built sample stage in which an angular mismatch (both polar and azimuthal) up to $\sim$5 $^{\rm o}$could be possible with respect to a veritable LMR. The situation is similar when all the electrodes were fully painted across the width (R_E, Figure S4c), but the angular mismatch seems even larger than the previous measurements. Note that these positive MR

regions completely disappear when the azimuthal magnetic fields (B_x and B_y) with proper values are turned on to overcome the angular mismatch, see the green curve ($R_{E'}$) in Figure S4c. This field refinement also suppresses the small upturn at high field as mentioned in the maintext. It is interesting to compare the field dependence of $R_{E'}$ to R_B. One clearly sees that both show negative MR under field and saturate to comparable values at high field, but R_B drops much faster that $R_{E'}$. All these measurements manifest an intrinsic negative LMR in $TaAs_2$, although the current-jetting effect could play some role if contacts are not properly made.

---

[1] Luo, Y. *et al.* Electron-hole compensation effect between topologically trivial electrons and nontrivial holes in NbAs. *Phys. Rev. B* **92**, 205134 (2015).

[2] Ren, Z., Taskin, A. A., Sasaki,S., Segawa, K., & Ando, Y. Large bulk resistivity and surface quantum oscillations in the topological insulator Bi_2Te_2Se. *Phys. Rev. B* **82**, 241306 (2010).

[3] Xiong, J. *et al.* High-field Shubnikov-de Haas oscillations in the topological insulator Bi_2Te_2Se, *Phys. Rev. B* **86**, 045314 (2012).

[4] Zhao, Y. *et al.* Anisotropic Fermi Surface and Quantum Limit Transport in High Mobility Three-Dimensional Dirac Semimetal Cd_3As_2. *Phys. Rev. X* **5**, 031037 (2015).

[5] Wang, K., Graf, D., Li, L., Wang, L., & Petrovic, C. Anisotropic giant magnetoresistance in $NbSb_2$. *Sci. Rep.* **4**, 7328 (2014).

[6] Cai, P. L. *et al.* Drastic Pressure Effect on the Extremely Large Magnetoresistance in WTe_2: Quantum Oscillation Study. *Phys. Rev. Lett.* **115**, 057202 (2015).

[7] Soluyanov, A. A. *et al.* Type-II Weyl semimetals. *Nature* **527** 495 (2015).

[8] Fu, L. & Kane, C. L. Topological insulators with inversion symmetry. *Phys. Rev. B* **76**, 045302 (2007).

[9] Autès, G. *et al.* A novel quasi-one-dimensional topological insulator in bismuth iodide β-Bi_4I_4. *Nature Mater.* Published online.

[10] Yoshida, K, A Geometrical Transport Model for Inhomogeneous Current Distribution in Semimetals under High Magnetic Fields. *J. Phys. Soc. Jpn.* **40**, 1027 (1976).

[11] Ueda, Y., & Kino, T. Anisotropy of the Apparent Resistivity in High-Purity Aluminium Single Crystals in Longitudinal Magnetic Fields. *J. Phys. Soc. Jpn.* **48**, 1601 (1980).

[12] Arnold, F. *et al.* Negative magnetoresistance without well-defined chirality in the Weyl semimetal TaP. Preprint at http://arxiv.org/abs/1506.06577 (2015).
